# A Systematic Review of Metabolite-to-Drug Ratios of Pharmaceuticals in Hair for Forensic Investigations

**DOI:** 10.3390/metabo11100686

**Published:** 2021-10-06

**Authors:** Karen Rygaard, Kristian Linnet, Sys Stybe Johansen

**Affiliations:** Section of Forensic Chemistry, Department of Forensic Medicine, Faculty of Health and Medical Sciences, University of Copenhagen, Frederik V’s Vej 11, 2100 Copenhagen, Denmark; kristian.linnet@sund.ku.dk (K.L.); sys.johansen@sund.ku.dk (S.S.J.)

**Keywords:** forensic science, hair analysis, head hair, pharmaceuticals, metabolite ratio

## Abstract

After ingestion, consumed drugs and their metabolites are incorporated into hair, which has a long detection window, ranging up to months. Therefore, in addition to conventional blood and urine analyses, hair analysis can provide useful information on long-term drug exposure. Meta-bolite-to-drug (MD) ratios are helpful in interpreting hair results, as they provide useful information on drug metabolism and can be used to distinguish drug use from external contamination, which is otherwise a limitation in hair analysis. Despite this, the MD ratios of a wide range of pharmaceuticals have scarcely been explored. This review aims to provide an overview of MD ratios in hair in a range of pharmaceuticals of interest to forensic toxicology, such as antipsychotic drugs, antidepressant drugs, benzodiazepines, common opiates/opioids, etc. The factors influencing the ratio were evaluated. MD ratios of 41 pharmaceuticals were reported from almost 100 studies. MD ratios below 1 were frequently reported, indicating higher concentrations of the parent pharmaceutical than of its metabolite in hair, but wide-ranging MD ratios of the majority of pharmaceuticals were found. Intra- and interindividual differences and compound properties were variables possibly contributing to this. This overview presents guidance for future comparison and evaluation of MD ratios of pharmaceuticals.

## 1. Introduction

Hair analysis can provide information regarding long-term drug exposure in addition to the conventional analyses of blood and urine analysis. Hair is a stable matrix with a long detection window, ranging up to months, depending on the length of the hair strands [1]. Therefore, drug concentrations in hair contribute to information about previous drug consumption, which is of interest in the field of forensic toxicology.

After administration, drugs are absorbed, distributed and metabolized in the body, depending on the administration route. During metabolism, the drugs are chemically degraded by enzymes, and the presence of metabolites can be used as evidence of active intake [2,3]. The drugs and their metabolites are incorporated into the hair. Various mechanisms for drugs entering hair have been proposed, but the precise mechanisms involved remain unclear. Proposed mechanisms include incorporation of drugs by passive diffusion from blood capillaries into the growing hair cells, incorporation from deep skin compartments during hair shaft formation, or deposition by diffusion from sweat or sebum secretions into the completed hair shaft [3]. Hair from the posterior vertex of the scalp grows 1 cm per month on average, and by dividing hair samples collected from that area into specified segments, drug consumption for months can be tracked. Nevertheless, hair analysis results must be interpreted with caution because variables such as age, drug metabolism, external contamination, hair melanin content, cosmetic hair treatment, and interindividual variability may influence them [4]. Differentiation between external contamination and incorporation of drugs through ingestion is a general issue in hair analysis and is of particular concern when interpreting the results [5]. It has been proposed that the detection of relevant metabolites and calculation of metabolite-to-drug (MD) ratios can minimize misinterpretation [6,7].

MD ratios in blood and urine are commonly reported in the literature and have been used to evaluate short-term abstinence in postmortem cases as well as acute intoxication, as these are dependent on sampling and dosing time [8]. In contrast, MD ratios in hair represent the cumulative amount of drugs and metabolites, as these are deposited in hair from blood and sweat during metabolism. Therefore, MD ratios in hair provide useful information on drug metabolism and can be used to distinguish drug use from external contamination, and are thus a helpful tool to interpret hair results [9]. External contamination is a major problem, especially when evaluating drugs of abuse in hair, but contamination with pharmaceuticals can also be a problem when differentiating a single intake in terms of intoxication from a long-term intake.

MD ratios of drugs of abuse in hair have frequently been reported [10,11,12,13], but the literature is still very limited regarding pharmaceuticals in hair. Despite this, a wide range of pharmaceuticals is commonly encountered in postmortem and forensic toxicology cases [14,15]. The limited information on such drugs, their metabolites, and the related MD ratio makes it difficult to interpret and compare hair analysis results and to eventually evaluate drug intake. Therefore, a comprehensive overview of the MD ratios of pharmaceuticals in hair is needed.

The objective of this systematic review is to give an overview of MD ratios in hair for pharmaceuticals of interest in forensic toxicology such as antipsychotic drugs, antidepressant drugs, benzodiazepines, common opiates/opioids, etc. In addition, factors influencing the MD ratio will be evaluated.

## 2. Methods

### 2.1. Information Sources and Search Strategy

This systematic review was performed according to the Preferred Reporting Items for Systematic Reviews and Meta-Analyses (PRISMA) guidelines [16,17]. The review was not registered and a review protocol was not prepared. Relevant studies were identified by searching the PubMed (National Library of Medicine 1966 to present) and Embase (1974 to present) electronic databases. The last search was performed on March 18 2021. The searches were refined using the following MeSH terms and text words: drug OR pharmaceutical AND hair AND concentration OR determination AND metabolite OR “degradation product” AND analysis. No filters were used for the special study design. Additionally, the reference list of each included study was checked in order to identify further studies missed during the initial search.

### 2.2. Exclusion Criteria

Title and abstracts were screened based on the following exclusion criteria:

- Studies not written in English;
- Reviews, letters, book chapters, or conference abstracts;
- Studies not of interest in forensic toxicology, e.g., cancer-related studies;
- Studies not concerning the analysis of authentic human head hair;
- Studies on hair from children ≤3 years old or unreported age;
- Studies not concerning antipsychotics, antidepressants, benzodiazepines, opioids/opiates (Except for morphine and codeine. See in Section 2.3);
- Studies not concerning other pharmaceuticals of interest in forensic toxicology, such as ketamine and methylphenidate;
- Studies on illegal drugs (New Psychoactive Substances (NPS)) or non-marketed drugs were excluded;
- Studies on compounds that cannot or have not been quantified in authentic hair (e.g., ethanol).

If even one of these criteria applied, the study was excluded from this review.

### 2.3. Study Selection

**Figure 1:**
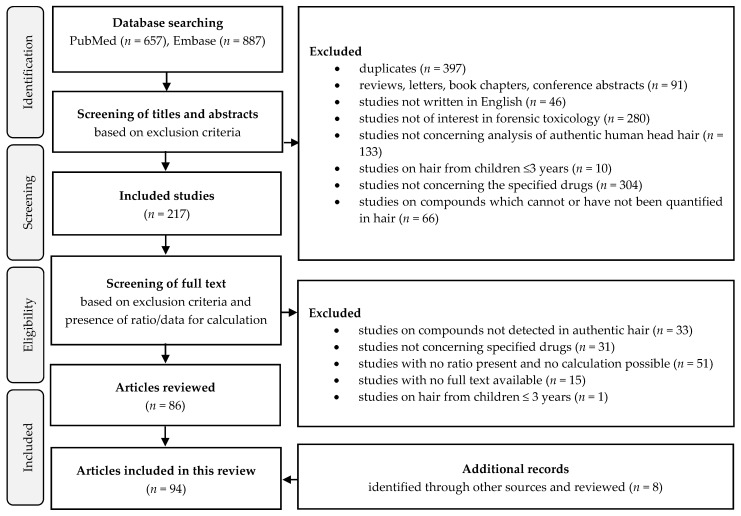
Flow chart of the study selection process developed according to PRISMA guidelines.

A total of 94 studies were included in this review. The study selection process is shown in a flow chart (Figure 1) and prepared according to the PRISMA guidelines. Studies with reported MD ratios or studies in which the calculation of MD ratios were possible were included. The MD ratios of drugs of abuse that are not legal pharmaceuticals and their metabolites were excluded from the present review as they have previously been explored in detail [10,11,12,13]. In addition, non-marketed pharmaceuticals were excluded. The MD ratios of morphine and codeine were omitted from the present review because it was difficult to distinguish therapeutic use from abuse and thus differentiate the ratios after morphine, codeine, and heroin intake.

### 2.4. Data Collection

Information on MD ratios of each pharmaceutical was obtained directly from the studies, from the calculation of the reciprocal value of reported drug-to-metabolite ratios, or calculated from visible and unmerged drug and metabolite concentrations in studies where no ratio was reported. Only concentrations above the lower limit of quantification or below the upper limit of quantification were included. The calculated MD ratio range was reported in this paper, and for studies with >10 included individuals or segments, percentiles were also reported. In addition, the mean and median MD ratios were calculated, if possible. Studies with children aged ≥ 4 years were included and the term children was used for individuals aged 4–9 years. MD ratios of pharmaceuticals and metabolites, which both can be ingested as the main drug, were excluded if intake of both was specified. This applied to diazepam and temazepam, among others.

## 3. Results and Discussion

MD ratios are helpful in interpreting hair results, as they provide useful information on drug metabolism and can be used to distinguish drug use from external contamination [7]. Despite this, the literature is still very limited regarding pharmaceuticals in hair. In order to obtain a sufficient amount of data, MD ratios reported in the literature together with MD ratios calculated from reported concentrations were included in this review. MD ratios for 41 pharmaceuticals from 94 studies have been included and summarized in Table 1. The table contains information on the MD ratios of the pharmaceuticals reported in each study, including the range and/or 10–90 percentiles of the MD ratios together with the median and mean MD ratios of each pharmaceutical, based on the information available. In addition, the associated case description, number of included individuals, information on the segmental analysis together with the total number of analyzed segments, and instrumental method for each study are included in the table. For pharmaceuticals with MD ratios reported by more than one study, the values were summarized with a combined ratio range representing the lowest and highest reported ratios, based on ranges and percentiles, as well as the reported median ratios presented as a range from the lowest reported median to the highest reported median. Percentiles of MD ratios were calculated for studies with more than 10 subjects/segments. For studies with only percentiles reported, this was used as the range of ratio. The MD ratios were summarized in this manner due to the inconstancy in the published MD ratios.

### 3.1. MD Ratios in Hair

Table 1 shows that MD ratios were most frequently reported for benzodiazepines and opioids/opiates, whereas reports on MD ratios of antidepressant and antipsychotic drugs were more limited. MD ratios of diazepam, ketamine, and methadone were reported from more than 14 studies each, while MD ratios of each antidepressant and antipsychotic pharmaceutical were reported from a maximum of seven studies.

**Figure 2:**
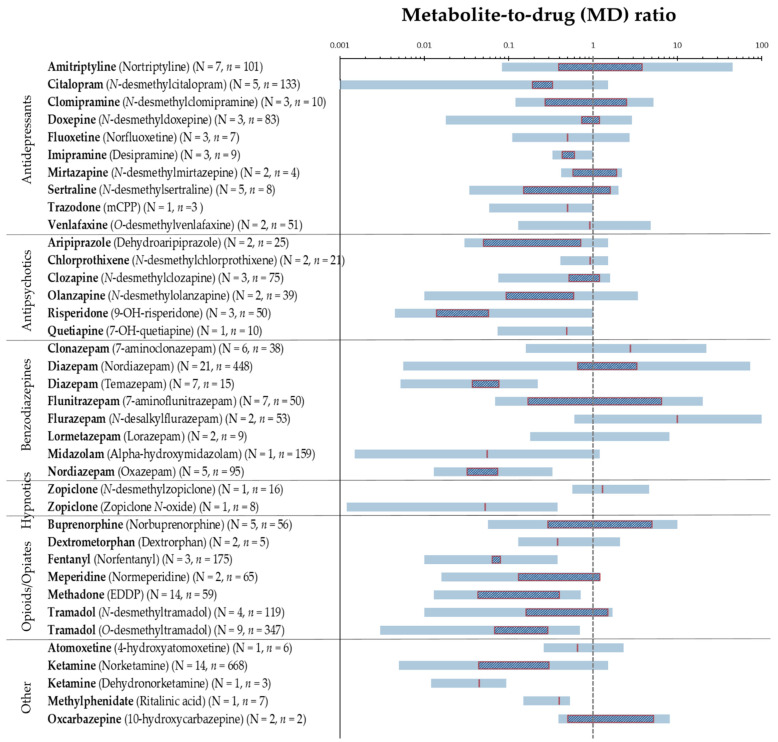
Visualization of MD ratios of 34 frequently reported pharmaceuticals, with N indicating the number of studies and n the number of individuals. Blue horizontal lines represent the MD ratio range from the lowest reported value to the highest. Red outlined squares represent range from the lowest reported median to highest, red vertical lines are cases with only one median value reported, and no line implies no reported median values. A logarithmic scale is used on the x-axis. The vertical dashed grey line indicates a MD ratio of 1.0. Cases with N = 1 and *n* = 1 were excluded.

Overall, MD ratios have been reported in the range from <0.005 to 110 based on all 41 pharmaceuticals, and for many of the included pharmaceuticals, wide-ranging ratios were seen both within and between studies. MD ratios of frequent pharmaceuticals from Table 1 are visualized in Figure 2, representing the summarized (combined) ratios.

Figure 2 illustrates the wide-ranging MD ratios of commonly reported pharmaceuticals based on the lowest and the highest reported values in the literature. In addition, it shows that median MD ratios below 1 were frequently reported, indicating that the parent drug concentration in hair exceeds that of its metabolites. There may be several explanations behind MD ratios below 1 being frequently found, including the differences in the incorporation into hair or the metabolism of the parent pharmaceutical relative to its metabolite. Various mechanisms for drugs entering the hair have been proposed and the properties of the incorporated drugs and metabolites, as well as the physical and physiological characteristics of the individual, may strongly influence which mechanism will dominate and thus the amount incorporated, the incorporation site and consequently the MD ratio [3]. The properties of the drugs and metabolites affecting the incorporation into hair include lipophilicity (logP), the basicity (pKa), melanin affinity, the half-life of elimination, and blood concentration. In general, lipophilic and basic compounds are better accumulated in pigmented hair than acidic compounds, which have been found in lower concentrations in hair [3]. This can explain the MD ratios less than 0.53 (*n* = 10, see Table 1) reported for methylphenidate and its more acidic metabolite ritalinic acid. MD ratios below 1 were primarily reported for several pharmaceuticals, including fentanyl, ketamine, methadone, and tramadol. This is in accordance with the higher parent drug concentrations reported for these pharmaceuticals by Musshoff et al. [89]. Similar physical and chemical properties were observed for many pharmaceuticals and their metabolites, as being the case for amitriptyline with pKa 9.4 and logP 4.9 and its metabolite nortriptyline with pKa 9.7 and logP 3.9 [110], and thus, the MD ratios were not found to be strongly affected by the small differences in lipophilicity or pKa values. Yet, the formation of metabolites by demethylation leading to decreased lipophilicity of the metabolite compared with the parent pharmaceutical can point to less effective incorporation and thus MD ratios below 1. This applies to most of the antidepressant pharmaceuticals, especially citalopram, doxepine, imipramine, and sertraline and for the antipsychotic pharmaceuticals, especially clozapine and olanzapine. In contrast, in plasma, the steady state concentrations of these metabolites have been found to be rather equal or higher than of the parent pharmaceuticals, giving MD ratios equal to or above 1 in plasma [23]. However, MD ratios in plasma and other fluid matrices are dependent on sampling and dosing time and represent a snapshot of the drug metabolism, which should always be considered. This contrasts MD ratios in hair representing the cumulative amount and the entire time course of drug metabolism [111].

MD ratios in hair above 1 were primarily reported for amitriptyline, buprenorphine, clomipramine, clonazepam, diazepam (with metabolite nordiazepam), flunitrazepam, flurazepam, oxcarbazepine (with metabolite 10-hydroxycarbazepine), and zopiclone (with metabolite *N*-desmethylzopiclone). A reason for this can be the longer elimination half-life of these metabolites compared with their parent pharmaceuticals [112,113]. For clonazepam and flunitrazepam, the stronger basicity of the amino-metabolites can account for the higher incorporation of the metabolites [41]. Despite this, MD ratios below 1 were also reported for these pharmaceuticals, indicating that the MD ratios were also highly affected by other variables, including interindividual differences.

### 3.2. Factors Affecting the MD Ratios in Hair

#### 3.2.1. Inter- and Intraindividual Differences

Interindividual differences may arise from differences in drug metabolism, hair color, cosmetic hair treatment, hair growth rate, the content of melanin in hair, sex, age, ethnicity, etc. Several studies have investigated the influence of interindividual differences on the MD ratio in hair. For instance, Papaseit et al. [92] found fluctuations in plasma concentrations after administering the same atomoxetine dose to different individuals and attributed this to interindividual differences in metabolism, including those due to the genetic polymorphism of CYP2D6, resulting in poor and extensive metabolizers. This can explain the differences in the reported MD ratios in hair ranging from 0.26 to 2.3 for atomoxetine and its metabolite 4-hydroxyatomoxetine (*n* = 6, Table 1). Other studies have likewise investigated the differences in metabolism due to the genetic polymorphisms of CYP2D6 and their influence on the MD ratios [85,86,88]. Johansen et al. [85] revealed a decreasing trend in the *O*-desmetyltramadol to tramadol ratio between extensive metabolizers of CYP2D6 (mean ratio: 0.30, *n* = 8), intermediate metabolizers of CYP2D6 (mean ratio: 0.21, *n* = 8), and the poor metabolizer of CYP2D6 (mean ratio: 0.072, *n* = 1), but an increasing trend of the *N*-desmetyltramadol to tramadol ratio in the same phenotype groups (mean ratio: 0.27, 0.34, and 1.5, respectively). The same trends were observed by Yu et al. [86], and this is a reasonable explanation for the observed variations in the reported ratios. The MD ratios of tramadol and its metabolites were also reported in the same ranges from other studies but without considering drug metabolism [20,28,87,91]. Furthermore, the incorporation of tramadol from sweat and the wash-out effect should also be considered when evaluating MD ratios. Krumbiegel et al. [27] presented drug incorporation via sweat as a justification for the observed variations in MD ratios. They found that differences in the intensity of sweating as well as possible higher drug incorporation via sweat after methadone ingestion due to heavy perspiration to be plausible reasons for the variations in MD ratios. Increased perspiration is a common side effect of both methadone, tramadol, and many antipsychotics and antidepressants, and in such cases, concomitant drug intake may influence the ratios. In addition, the concomitant consumption of psychotropic medication metabolized by the same isoenzymes affects the drug metabolism and thus the MD ratio [92]. Musshoff et al. [89] reported strong inhibition of the enzyme CYP2B6 by selective serotonin reuptake inhibitors. CYP2B6 metabolizes ketamine to its metabolite norketamine, and concomitant drug intake may be a reason for varying MD ratios of ketamine and norketamine, which were reported from 0.010 to 1.3 in hair (*n* = 654, see Table 1).

For most of the MD ratios reported in the present review, the segment in which the MD ratio was found was not taken into account. Klys et al. [26] reported decreasing MD ratios of clomipramine and its metabolite *N*-desmethylclomipramine in the segments situated closer to the hair bulb, indicating higher levels of metabolite in the segments closest to the scalp (proximal segments) and thus intraindividual variations in the MD ratio. This explains why MD ratios both below and above 1 have been reported. Similar findings were observed for methadone, quetiapine, risperidone, and tramadol [27,31,39,85]. Other studies revealed only small variations in the MD ratios in all hair segments, from the proximal end to the distal end, for patients with a presumed long-term intake of either aripiprazole, chlorprothixene, or clozapine [31,34,35,63]. Similar results were reported for a single dose study of zopiclone in 0.5 cm segments up to four months after ingestion by Hansen et al. [63]. Total intraindividual variations in MD ratios of less than 20% of zopiclone and aripiprazole have been reported [34,63]. However, these studies did not elaborate further on factors such as hair pigmentation or cosmetic and physical hair treatment, which can influence the amount of pharmaceuticals or metabolites measured in hair.

#### 3.2.2. Hair Characteristic

Kronstrand et al. [36] found significantly different mean MD ratios of clozapine and its metabolite *N*-desmethylclozapine in pigmented (1.2, *n* = 12) and non-pigmented (0.93, *n* = 11) hair, indicating better incorporation of the metabolites into pigmented hair (See Table 1). Higher nordiazepam than diazepam concentrations was also reported in black hair after a single dose of diazepam (*n* = 8) [47]. In contrast, Wang et al. [31] presented MD ratios of aripiprazole and its metabolite dehydroaripiprazole in black hair, which were one-tenth of the other reported MD ratios, indicating better incorporation of the parent pharmaceutical in black hair (pigmented hair) [34]. Licata et al. [20] reported higher levels of the metabolite nortriptyline compared to amitriptyline for patients with hair that is gray or subjected to cosmetic treatment, which can explain the MD ratios greater than 1 mentioned earlier. Likewise, olanzapine concentrations were found to be low in gray hair [37]. These observations reaffirm the influence of hair characteristics on the MD ratios of some pharmaceuticals. Fernández et al. [114] determined the influence of hair color and melanin content on hair analysis by measuring the antipsychotic drugs chlorpromazine, haloperidol, olanzapine, quetiapine, and risperidone (+ metabolites) in the melanin and protein fractions of collected hair samples. They found that hair melanin had a higher affinity for the five antipsychotic drugs compared to the protein fraction but that the results were influenced more by the effects of biochemical individuality and less by hair color. Unfortunately, in forensic investigations, information on these variables and on the actual intake are often limited, which may complicate the interpretation of the hair analysis results.

#### 3.2.3. Drug Intake

MD ratios may be a helpful tool to differentiate between actual intake and external contamination. Madry et al. [88] investigated MD ratios in individuals with a passive exposure working at a tramadol production company. They found that the *O*-desmethyltramadol to tramadol ratios in tramadol users were significantly higher than in subjects with passive exposure. They suggested an MD ratio of tramadol and its metabolite *O*-desmethyltramadol of 0.021 and an MD ratio of tramadol and *N*-desmethyltramadol of 0.037 to be used to distinguish drug ingestion from external contamination. This distinction is a major problem especially for drugs of abuse, whereas contamination with pharmaceuticals to a greater extent is a problem when distinguishing a single ingestion in terms of intoxication from long-term intake. Such differentiations are only possible with segmental hair analysis. In the present review, no trends were found indicating lower or higher MD ratios in single-dose compared with long-term use. However, only a few of the included studies were controlled single-dose or long-term use studies. For pharmaceuticals with a short elimination half-life, MD ratios above 1 might be expected after single dose intake, whereas MD ratios after long-term use may rather be dependent upon other factors such as compound properties due to steady state plasma concentrations. Despite this, Xiang et al. [106] reported very high ketamine concentrations and low norketamine concentrations in the hair of drug abusers after occasional use of ketamine, indicating the great influence on MD by other factors. In many of the studies, it was not clear whether the pharmaceuticals were used or abused, and in general it is difficult to distinguish therapeutic use from abuse. No clear differences were observed in the MD ratios for drug use cases compared with drug abuse cases. In addition, Papaseit et al. [92] found no linear relationship between the administered daily dose, daily dose per kg weight, and the MD ratio of atomoxetine and its metabolite 4-hydroxyatomoxetine in hair segments from any of the included individuals (*n* = 6).

Another factor to be considered is metabolites serving as prescription drugs themselves, including 9-OH-risperidone, lorazepam, nordiazepam, norfluoxetine, nortriptyline, oxazepam, oxymorphone, and temazepam. In addition, some pharmaceuticals, especially benzodiazepines, are formed from more than one parent pharmaceutical. In such cases, the MD ratios may be artificially elevated, which causes wide-range MD ratios and complicates the interpretation and comparison of MD ratios both within and between studies.

#### 3.2.4. Pre-Analytical Workflow and Analytics

Skopp et al. [115] reported a potential underestimation of buprenorphine and norbuprenorphine concentrations in hair and reversed concentration ratios if these compounds were recovered by acidic procedures. This indicates that the sample preparation procedure may also be a cause for the different MD ratios reported. Kintz [6] described the possibilities of postmortem incorporation of drugs into hair through blood, sweat, etc., demonstrating that determining accurate MD ratios also critically depends on effective washing procedures for the sufficient removal of external contamination [6,116]. Postmortem incorporation via sweat is critical because sweat may contain both the parent compound and the metabolite, complicating the interpretation of the determined concentrations in hair and MD ratios [19]. Other variables in the sample preparation process, including differences in sample collection, segmentation, and preparation of hair samples (wash procedure and the powering or cutting hair before extraction), may influence the MD ratio as well.

Table 1 highlights the frequent use of LC–MS as well as GC–MS methods for hair analysis. A benefit of using GC–MS is more robustness against the matrix effect. The matrix effects may suppress or enhance the analyte signal during analysis, leading to inaccurate analysis results. LC–MS methods are more highly influenced by the matrix effect, and in such cases, the choice of internal standard is highly important to obtaining accurate analysis results. Higher accuracies are associated with coeluting isotope-labeled internal analogs, as they allow for better correction. More than 50% of the MD ratios were reported from studies that did not involve the use of isotope-labeled internal analogs for either the pharmaceutical or the metabolite, and this too can explain the wide-ranging MD ratios.

Finally, better comparability among different methods and laboratories can be established from external proficiency testing, which unfortunately is still limited for many pharmaceuticals. Thus, we encourage researchers in the field of forensic hair analysis to report MD ratios in hair in order to obtain more reference values and to better understand the factors influencing the ratios. The overview of MD ratios in this review provides guidance for evaluating MD ratios in the future.

### 3.3. Limitations

This review is based on MD ratios reported or calculated from other studies; therefore, the information available was limited. In addition, inconsistency in the way the ratios were reported, varieties in hair and segment length, and the number of subjects in the different studies complicated the comparison of the MD ratios. Due to the inconsistency in the way the MD ratios were reported, this review showed MD ratio ranges based on the minimum and maximum values together with median intervals, including the lowest reported median and the highest median, if available. MD ratio ranges found only from minimum and maximum values contribute to the wide-ranging MD ratio ranges observed. Therefore, MD ratios reported as 10–90 percentile ranges would be preferred as these exclude the extreme values and thus improve the range for interpretation.

As seen from Table 1, MD ratios have been reported for different types of cases, including deceased individuals, individuals in treatment, and individuals with drug abuse. In many of the postmortem cases, it was not clear whether the intake of the particular pharmaceutical led to intoxication and was the cause of death or if the pharmaceutical were taken regularly. Such differentiations are only possible with segmental analysis, which has only been occasionally published in the studies. These limitations minimize the possibilities to further interpret MD ratios across different case types.

Furthermore, it was not possible in all cases to identify whether metabolites served as pharmaceuticals themselves or were metabolized from multiple pharmaceuticals, leading to discrepancies in the MD ratios. The wide range of reported MD ratios of the pharmaceuticals highlighted in the present review may limit the interpretation of MD ratios, and, therefore, MD ratios need to be reported in a more consistent way, and need to be studied further.

## 4. Conclusions

The present review provides a comprehensive overview of the metabolite-to-drug (MD) ratios of pharmaceuticals in hair, a topic that is very limited in the literature. MD ratios of 41 pharmaceuticals from 94 studies were reported. The most frequently reported were MD ratios below 1, indicating that the parent pharmaceutical concentration in hair exceeded metabolite concentration in most cases. In addition, the review highlights that MD ratios of a majority of the pharmaceuticals were wide-ranging. Among others, intra- and interindividual differences and compound properties were variables that could possibly have contributed to this. This overview of MD ratios and medians can provide helpful guidance for the comparison and evaluation of MD ratios of pharmaceuticals in hair reported in future research.

## Figures and Tables

**Table 1:** Overview of metabolite-to-drug (MD) ratios of pharmaceuticals in hair from the literature. Data are sorted by indication group, pharmaceutical, and year of publication. The combined row (bold and italic text) summarizes the reported data for each pharmaceutical with a ratio interval and a median ratio presented as a range from the lowest reported median to the highest reported median, if it appears in more than one study. Gray shades indicate self-calculated ratios from published concentrations.

Pharmaceutical (Metabolite)	*MD Ratio*				No. of Subjects	No. of Seg. Per Subject (Seg. Length)	Total No. of Seg. (Whole Strand)	Case Description	Analytical Method	Reference
	Range	10–90 PCTL ^(A)^	Mean	Median						
**ANTIDEPRESSANTS**										
**Amitriptyline** (Nortriptyline)	0.083–5.5	0.15–5.0 ^(B)^	1.4	1.1	45	2 (2 cm) or full length	42 (24)	PM cases, presumed intake	LC–MS/MS	Methling (2020) [18]
	0.63–45	-	14	3.8	2	2–5 (1 cm)	7	AD users, presumed intake	LC–MS/MS	Fernández (2016) [19]
	0.24–3.2	-			24	1 (3 cm)	24	Headache patients	LC–MS/MS	Licata (2016) [20]
	2.4	-	2.4	-	1	1 (9 cm)	1	Patient under treatment with psychoactive drugs	LC–MS/MS	Fisichella (2014) [21]
	0.97–3.3	-	2.2	2.2	2	1 (1–3)	2	PM cases	LC–HRMS	Nielsen (2010) [22]
	0.1–2.6	0.17–0.80	0.56	0.39	25	1 (3 cm)	25	Psychiatric patients, presumed intake	GC–MS	Pragst (1997) [23]
	0.88–2.9	-	1.9	1.9	2	Unspecified	≥2	Patients under long-term treatment	GC–MS	Ishiyama (1983) [24]
	***0.083–45***			***0.39–3.8***	***101***		***103 (24)***			***Combined***
**Citalopram** (*N*-desmethyl-citalopram)		0.066–0.87 ^(B)^	0.39	0.33	108	2 (2 cm) or full length	136 (40)	PM cases, presumed intake	LC–MS/MS	Methling (2020) [18]
	0.13–1.5	0.14–0.62	0.33	0.23	3	4–10 (1 cm)	20	AD users, presumed intake	LC–MS/MS	Fernández (2016) [19]
	0.35–0.88	-	-	-	16	1 (3 cm)	16	Headache patients	LC–MS/MS	Licata (2016) [20]
	0.00069–0.67	-	0.25	0.19	5	1 (9 cm)	5	PM cases and patients under treatment with psychoactive drugs	LC–MS/MS	Fisichella (2014) [21]
	0.76	-	0.76	-	1	1 (2 cm)	1	PM case, polydrug intoxication	GC–MS	Wille (2009) [25]
	***0.00069–1.5***			***0.19–0.33***	***133***		***178 (40)***			***Combined***
**Clomipramine** (*N*-desmethyl-clomipramine)	1.8–5.2	1.8–5.2 ^(B)^	3	2.5	4	2 (2 cm) or full length	4 (2)	PM cases, presumed intake	LC–MS/MS	Methling (2020) [18]
	0.75–2.2	-	1.7	2.1	1	3 (4 cm)	3	PM case, under treatment, alcohol abuse	LC–MS	Klys (2005) [26]
	0.12–0.86	-	0.37	0.27	5	1 (3 cm)	5	Psychiatric patients, presumed intake	GC–MS	Pragst (1997) [23]
	***0.12–5.2***			***0.27–2.5***	***10***		***12 (2)***			***Combined***
**Doxepine** (*N*-desmethyldoxepine)	0.018–2.9	0.14–2.3 ^(B)^	1.2	1.2	76	2 (2 cm) or full length	98 (27)	PM cases, presumed intake	LC–MS/MS	Methling (2020) [18]
	0.85	-	0.85	-	1	1 (6 cm)	1	PM cases, drug abuse history	LC–MS/MS	Krumbiegel (2016) [27]
	0.33–1.4	-	0.81	0.73	6	1 (3 cm)	6	Psychiatric patients, presumed intake	GC–MS	Pragst (1997) [23]
	***0.018–2.9***	**-**		***0.73–1.2***	***83***		***105 (27)***			***Combined***
**Fluoxetine** (Norfluoxetine)	0.15	-	0.15	-	1	1 (2 cm)	1	Suspected DFC case	LC–MS/MS	Wang (2018) [28]
	0.11–0.67	0.12–0.61	0.38	0.50	2	4–6 (1 cm)	10	AD users, presumed intake	LC–MS/MS	Fernández (2016) [19]
	0.93–2.7	-	-	-	4	1 (3 cm)	4	Headache patients	LC–MS/MS	Licata 2016 [20]
	***0.11–2.7***			***0.50***	***7***		***15***			***Combined***
**Imipramine** (Desipramine)	0.36–0.8	-	0.62	0.6	5	1 (3 cm)	5	Psychiatric patients, presumed intake	GC–MS	Pragst (1997) [23]
	0.85	-	0.85	-	1	Unspecified	≥1	PM case	GC–MS + LC	Couper (1995) [29]
	0.33–1.0	-	0.59	0.43	3	Unspecified	≥3	Patients under long-term treatment	GC–MS	Ishiyama (1983) [24]
	***0.33–1.0***			***0.43–0.6***	***9***		**≥*9***			***Combined***
**Mirtazapine** (*N*-desmethyl-mirtazapine)	0.42–0.90	-	0.60	0.58	2	3 (1 cm)	6	Children administered mirtazapine without consent	LC–MS/MS	Kintz (2021) [30]
	1.6–2.2	-	1.9	1.9	2	1 (3 cm)	2	Headache patients	LC–MS/MS	Licata (2016) [20]
	**0.42–2.2**			**0.58–1.9**	**4**		**8**			***Combined***
**Sertraline** (*N*-desmethylsertraline)	1.7	-	1.7	-	1	1 (2 cm)	1	Schizophrenic patients under treatment, in compliance	LC–MS/MS	Wang (2019) [31]
	0.18–0.89	-	0.50	0.50	1	6 (2 cm)	6	Child suspected accidental intoxication	LC–MS/MS	Marchei (2016) [32]
	0.79–2.0	-	-	-	4	1 (3 cm)	4	Headache patients	LC–MS/MS	Licata (2016) [20]
	0.034–0.16	-	0.12	0.15	1	3 (3 cm)	3	AD and anxiolytic drug consumption during pregnancy	LC–MS/MS	Pichini (2016) [33]
	0.83–1.8		1.4	1.6	1	3 (1.5–2 cm)	3	PM case, polydrug intoxication	GC–MS	Wille (2009) [25]
	***0.034–2.0***			***0.15–1.6***	***8***		***17***			***Combined***
**Trazodone** (mCPP (M-chloro-phenylpiperazine))	0.059–1.0	0.088–0.50	0.37	0.50	3	1–10 (1 cm)	13	AD users, presumed intake	LC–MS/MS	Fernández (2016) [19]
**Venlafaxine** (O-desmethyl-venlafaxine)	-	0.13–3.5	1.5	0.92	45	2 (2 cm) or full length	50 (20)	PM case, presumed intake	LC–MS/MS	Methling (2020) [18]
	1.2–4.8	-	-	-	6	1 (3 cm)	6	Headache patients	LC–MS/MS	Licata (2016) [20]
	***0.13–4.8***			***0.92***	***51***		***56 (20)***			***Combined***
**ANTIPSYCHOTICS**										
**Aripiprazole** (Dehydroaripiprazole)	0.21–1.5	0.3–1.3	0.76	0.72	16	1–6 (1 cm)	71	PM cases of psychiatric patients with presumed intake	LC–MS/MS	Rygaard (2020) [34]
	0.030–0.093	0.034–0.083	-	0.050	9	1–2 (2 cm)	11	Schizophrenic patients under drug treatment, in compliance	LC–MS/MS	Wang (2019) [31]
	***0.030–1.5***			***0.050–0.72***	***25***		***82***			***Combined***
**Chlorprothixene** (*N*-desmethyl-chlorprothixene)	-	0.41–1.5	-	0.93	20	3–6 (1 cm)	≥60	PM cases of psychiatric patients with presumed intake	LC–MS/MS	Günther (2018) [35]
	1.5	-	1.5	-	1	1 (2 cm)	1	Suspected DFC case	LC–MS/MS	Wang (2018) [28]
	***0.41–1.5***		***-***	***0.93***	***21***		***≥61***			***Combined***
**Clozapine** (*N*-desmethylclozapine)	-	0.075–1.3 ^(B)^	0.57	0.52	25	2 (2 cm) or full length	28 (11)	PM cases presumed intake	LC–MS/MS	Methling (2020) [18]
	0.52–1.5	0.77–1.4	-	1.1	27	1–3 (2 cm)	54	Schizophrenic patients under drug treatment, in compliance	LC–MS/MS	Wang (2019) [31]
	0.97–1.6 0.70–1.3	1.0–1.6 0.75–1.1	1.2 0.96	1.2 0.97	12 11	1 (2–7 cm) 1 (2–7 cm)	12 11	Patients in low-dose treatment, pigmented hair and non-pigmented hair	LC–MS/MS	Kronstrand (2007) [36]
	***0.075–1.6***			***0.52–1.2***	***75***		***105 (11)***			***Combined***
**Olanzapine** (*N*-desmethylolanzapine)	0.010–3.4	-	-	0.59	34	1–6 (1 cm) or full length (if <2 cm)	105	PM cases of psychiatric patients, presumed intake	LC–MS/MS	Günter (2020) [37]
	0.063–0.23	0.063–0.23	-	0.093	5	1–2 (2 cm)	7	Schizophrenic patients under drug treatment, in compliance	LC–MS/MS	Wang (2019) [31]
	***0.010–3.4***			***0.093–0.59***	***39***		***112***			***Combined***
**Risperidone** (9-OH-risperidone)	0.0045–0.093	0.0055–0.031	-	0.014	12	1–3 (2 cm)	27	Schizophrenic patients under drug treatment, in compliance	LC–MS/MS	Wang (2019) [31]
	-	0.0047–1 ^(B)^	0.17	0.058	35	2 (2 cm) or full length	30 (20)	PM cases, presumed intake	LC–MS/MS	Methling (2020) [18]
	0.012–1.0	-	0.31	0.023	3	1–4 (2–10 cm)	8	Psychiatric patients	LC–MS/MS	Schneider (2009) [38]
	***0.0045–1.0***			***0.014–0.058***	***50***		***65 (20)***			***Combined***
**Quetiapine** (7-OH-quetiapine)	0.074–1	0.12–0.91	0.50	0.49	10	1–6 (2 cm)	26	Patients under drug treatment	LC–MS/MS	Binz (2014) [39]
**BENZODIAZEPINES**										
**Alprazolam** (Alpha-hydroxyalprazolam)	0.056–0.062	-	0.059	0.059	1	3 (2 cm)	3	PM case, presumed intake, drug addiction	LC–MS/MS	Wang (2017) [40]
**Clonazepam** (7-aminoclonazepam)	0.16–17	-	-	2.8	33	1 (≤5 cm)	33	Individuals undergoing toxicological investigation	LC–MS/MS	Madry (2020) [41]
	82	-	82	-	1	Unspecified	≥1	Suspected drug abuser	LC–MS/MS	Shin (2019) [42]
	1.6	-	1.6	-	1	1 (2 cm)	1	Suspected DFC case	LC–MS/MS	Wang (2018) [28]
	3.7	-	3.7	-	1	1 (3 cm)	1	Headache patient	LC–MS/MS	Licata (2016) [20]
	22	-	22	-	1	Unspecified	≥1	DFC case	LC–MS/MS	Chéze 2005 [43]
	7.0	-	7.0	-	1	1 (2 cm)	1	Individuals under treatment	GC–MS	Negruz (2000) [44]
	***0.16–22***			***2.8***	***38***		***≥38***			***Combined***
**Delorazepam** (Lorezepam)	≤0.090	-	-	-	23	1 (3 cm)	23	Headache patients	LC–MS/MS	Licata (2016) [20]
**Diazepam** (Nordiazepam)	0.0056–26	-	-	1.6	293	1 (≤5 cm)	293	Individuals undergoing toxicological investigation	LC–MS/MS	Madry (2020) [41]
	-	0.26–2.9 ^(B)^	1.3	1.1	71	2 (2 cm) or full length	70 (36)	PM cases, presumed intake	LC–MS/MS	Methling (2020) [18]
	0.40	-	0.40	-	1	Unspecified	≥1	Individuals with suspected drug abuse	LC–MS/MS	Shin (2019) [42]
	0.96	-	0.96	-	1	1 (3–6 cm)	1	Driving licence regranting	LC–MS/MS	Lendoiro (2018) [45]
	0.58–1.6	-	1.0	0.98	2	1–3 (0.8–1.5 cm)	4	Suspected DFC case	LC–MS/MS	Wang (2018) [28]
	0.98	-	0.98	-	1	1 (3–6 cm)	1	PM case	LC–MS/MS	Morini (2017) [46]
	1.3–4.1	-	2.3	2.1	8	1 (2 cm)	8	Controlled single dose study, proximal seg. (1 month after intake) and	LC–MS/MS	Wang (2017) [47]
	0.77–1.1	-	0.91	0.88	6	1 (2 cm)	6	second seg.		
	6	-	6	-	1	1 (2 cm)	1	DFC case	LC–MS/MS	Kim (2016) [48]
	0.23–1.6	0.39–1.3	0.89	0.94	5	1–4 (1.5–8 cm)	10	PM cases	LC–MS/MS	Krumbiegel (2016) [27]
	0.30–2.2	-	-	-	3	1 (3 cm)	3	Headache patients	LC–MS/MS	Licata (2016) [20]
	2.0–2.8	-	2.4	2.5	1	3 (3 cm)	3	AD and anxiolytic drug consumption during pregnancy	LC–MS/MS	Pichini (2016) [33]
	1.1–1.1	-	1.1	1.1	1	2 (4 cm)	2	PM case	LC–MS/MS	Maublanc 2015 [49]
	0.31–5.5	-	2.1	0.66	5	1 (3 or 9 cm)	5	PM cases and patients under treatment with psychoactive drugs	LC–MS/MS	Fisichella (2014) [21]
	1.3–1.7	-	1.5	1.5	2	Unspecified	≥2	Patient following withdrawal treatment and individual with suspected drug abuse	LC–MS/MS	Lendoiro (2012) [50]
	0.67–5	-	2.8	3.3	5	Unspecified	≥5	Presumed drug use	LC–HRMS	Favretto (2011) [51]
	0.35–1.6	-	0.96	0.96	2	Unspecified	≥2	Individuals with suspected drug abuse	LC–MS/MS	Kim (2011) [52]
	0.35 0.27	- -	0.35 0.27	- -	1	Unspecified (10–35 cm)	≥1	Individuals with suspected drug abuse non-pigmented hair and pigmented hair	GC–MS	Lee (2011) [53]
	0.13–6.7	0.36–5.0	2.2	1.6	14	1–3 (1–3 cm)	16	PM cases and individuals undergoing toxicological investigation	LC–HRMS	Vogliardi (2011) [15]
	0.94–7.3	1.1–6.7	3.1	2.8	8	1–2 (0.5–3 cm)	10	PM cases undergoing toxicological investigation	LC–MS/MS	Miller (2008) [54]
	0.46–8	-	3.3	1.5	3	Unspecified	≥3	PM cases, presumed intake	LC–MS/MS	Ariffin (2007) [55]
	0.21–73	0.53–40	15	2.4	14	1 (3 cm)	14	PM cases, dead from a drug overdose	GC–MS	Yegles (1997) [56]
	***0.0056–73***			***0.66–3.3***	***448***		**≥*461 (36)***			***Combined***
**Diazepam** (Temazepam)	0.023	-	0.023	-	1	1 (0.8 cm)	1	Suspected DFC case	LC–MS/MS	Wang (2018) [28]
	0.048–0.090	-	0.066	0.064	4	1–4 (1.5–6 cm)	9	PM cases	LC–MS/MS	Krumbiegel (2016) [27]
	0.076–0.078	-	0.077	0.077	1	2 (4 cm)	2	PM case	LC–MS/MS	Maublanc 2015 [49]
	0.0052– 0.068	-	0.037	0.037	2	unspecified	≥2	Individuals with suspected drug abuse	LC–MS/MS	Kim (2011) [52]
	0.050–0.11	-	0.069	0.063	5	1 (1–3 cm)	5	PM cases and individuals undergoing toxicological investigation	LC–HRMS	Vogliardi (2011) [15]
	0.19	-	0.19	-	1	1 (3 cm)	1	PM cases undergoing toxicological investigation	LC–MS/MS	Miller (2008) [54]
	0.22	-	0.22	-	1	unspecified	≥1	PM cases, presumed intake	LC–MS/MS	Ariffin (2007) [55]
	***0.0052–0.22***			***0.037–0.077***	***15***		**≥*21***			***Combined***
**Flunitrazepam** (7-amino-flunitrazepam)	2.1–20	-	-	6.5	8	1 (≤5 cm)	8	Individuals undergoing toxicological investigation	LC–MS/MS	Madry (2020) [41]
	-	1.3–5.0 ^(B)^	3.2	3.1	3	2 (2 cm) or full length	4 (1)	PM cases, presumed intake	LC–MS/MS	Methling (2020) [18]
	-	0.51–8.3	-	3.07	22	3–6 (1 cm)	76	Individuals undergoing toxicological investigation, repeated use	LC–MS/MS	Zhuo (2020) [9]
	1.1	-	1.1	-	1	1 (3 cm)	1	DFC case	LC–MS/MS	Kim (2016) [48]
	1.1	-	1.1	-	1	unspecified	≥1	PM case	GC–MS	Negrusz (1999) [57]
	0.069–3.5	0.27–2.5	1.2	0.90	14	Whole strand	(14)	PM cases, polydrug abusers	GC–MS	Cirimele (1997) [58]
	0.11–0.27	-	0.18	0.17	1	3 (3–4 cm)	3	Individual with a suspected drug abuse	GC–MS	Cirimele (1996) [59]
	***0.069–20***			***0.17–6.5***	***50***		***93 (15)***			***Combined***
**Flurazepam** (2-hydroxyethyl-flurazepam)	0.033	-	0.033	-	1	1 (3 cm)	1	Headache patients	LC–MS/MS	Licata (2016) [20]
**Flurazepam** (*N*-desalkylflurazepam)	0.60–110	-	-	10	52	1 (≤5 cm)	52	Individuals undergoing toxicological investigation	LC–MS/MS	Madry (2020) [41]
	2.4	-	2.4	-	1	1 (3 cm)	1	Headache patients	LC–MS/MS	Licata (2016) [20]
	***0.60–110***			***10***	***53***		***53***			***Combined***
**Lormetazepam** (Lorazepam)	0.18–8	-	-	-	8	1 (≤5 cm)	8	Individuals undergoing toxicological investigation	LC–MS/MS	Madry (2020) [41]
	0.42	-	0.42	-	1	1 (3 cm)	1	Headache patients	LC–MS/MS	Licata (2016) [20]
	***0.18–8***			**-**	***9***		***9***			***Combined***
**Midazolam** (alpha-hydroxymidazolam)	0.0015–1.2	-	-	0.056	159	1 (≤5 cm)	159	Cases of interest in forensic toxicolgy	LC–MS/MS	Madry (2020) [41]
**Nordiazepam** (Oxazepam)	0.017–0.28	-	-	0.074	86	1 (≤5 cm)	86	Individuals undergoing toxicological investigation	LC–MS/MS	Madry (2020) [41]
	0.040–0.10	-	0.070	0.070	2	Full length (5–7 cm)	(2)	Individuals with past history depressive disorder	LC–MS/MS	Wiart (2020) [60]
	0.030	-	0.030	-	1	1 (0.8 cm)	1	Suspected DFC case	LC–MS/MS	Wang (2018) [28]
	0.33	-	0.33	-	1	1 (3 cm)	1	PM cases undergoing toxicological investigation	LC–MS/MS	Miller (2008) [54]
	0.013–0.056	-	0.034	0.032	5	Unspecified	≥5	Individuals with polydrug abuse	GC–MS	Kintz (1996) [61]
	***0.013–0.33***			***0.032–0.074***	***95***		***≥93 (2)***			***Combined***
**Triazolam** (1-hydroxymethyl-triazolam)	2.1	-	2.1	-	1	Hair shaved	(1)	PM case, individual with drug addiction	LC–MS	Toyo’oka (2001) [62]
**Triazolam** (4-hydroxy triazolam)	14	-	14	-	1	Hair shaved	(1)	PM case, individual with drug addiction	LC–MS	Toyo’oka (2001) [62]
**HYPNOTICS**										
**Zolpidem** (zolpidem phenyl 4carboxylic acid)	0.0018	-	0.0018	-	1	Unspecified	≥1	Individuals with suspected drug abuse	LC–MS/MS	Shin (2019) [42]
**Zopiclone** (*N*-desmethylzopiclone)	0.57–4.6	-	-	1.3	16	2–12 (0.5 cm)	56	Controlled single dose study	LC–MS/MS	Hansen (2020) [63]
**Zopiclone** (zopiclone *N*-oxide)	0.0012–0.38	-	-	0.053	8	2–12 (0.5 cm)	31	Controlled single dose study	LC–MS/MS	Hansen (2020) [63]
**OPIOIDS/OPIATES**										
**Buprenorphine** (Norbuprenorphine)	1.1	-	1.1	-	1	1 (3.5 cm)	1	PM case, drug abuse history	LC–MS/MS	Krumbiegel (2016) [27]
	- - -	1.7–5 ^(C)^ 3.3–10 ^(C)^ 1.4–10 ^(C)^	1.7 2.5 2.5	3.3 5 3.3	36	1–3 (3.9 ± 1.2 cm) (5.0 ± 2.1 cm) (4.5 ± 2.0 cm)	19 17 15	Individuals under treatment, proximal seg. and middle seg. and distal seg.	LC–MS	Belivanis (2013) [64]
	2.0–3.0	-	2.7	2.7	3	Unspecified	≥3	Presumed drug use	LC–HRMS	Favretto (2011) [51]
	0.49–2.9	-	1.9	1.5	5	Unspecified(1 cm)	≥5	Long history of buprenorphine abuse	GC–MS	Vincent (1999) [65]
	0.057–0.64	0.11–0.44	0.28	0.29	11	Unspecified	≥11	Drug abuse history (Heroin)	LC–ECD	Kintz (1994) [66]
	***0.057–10***			***0.29–5***	***56***		***≥71***			***Combined***
**Dextrometorphan** (Dextrorphan)	0.40	-	0.40	-	1	1 (12 cm)	1	Individuals with suspected drug abuse	LC–MS/MS	Kim (2014) [67]
	0.13–2.1	-	0.76	0.38	4	Whole strand	(4)	Individuals with suspected abuse	GC–MS	Kim (2004) [68]
	***0.13–2.1***			***0.38***	***5***		***1 (4)***			***Combined***
**Fentanyl** (Norfentanyl)	0.01–0.38	-	0.10	0.08	154	Full length (1–20 cm)	(154)	Suspected intake	LC–MS/MS	Salomone (2020) [69]
	0.031–0.15	0.034–0.096	0.069	0.064	20	Full length (1–20 cm)	(20)	Individuals using heroine	LC–MS/MS	Palamar (2019) [70]
	0.03	-	0.03	-	1	1 (3.5 cm)	1	PM case, drug abuse history	LC–MS/MS	Krumbiegel (2016) [27]
	***0.01–0.38***			***0.08–0.064***	***175***		***1 (174)***			***Combined***
**Meperidine** (Normeperidine)	0.016–0.39	-	0.17	0.13	5	1 (9–22 cm)	5	Individuals with suspected abuse	LC–MS/MS	Kim (2014) [67]
	-	-	1.40	1.21	60	Unspecified(1–5 cm)	≥71	Individuals with meperidine addiction	GC–MS	Min (1999) [71]
	***0.016–0.39***			***0.13–1.21***	***65***		***≥76***			***Combined***
**Methadone** (EDDP (2-ethyl-1,5-dimethyl- 3,3-diphenylpyrrolidine))	0.30	-	0.30	-	1	Full length	(1)	Individual undergoing methadone treatment program	GC–MS/MS	Rosado (2020) [72]
	0.063	-	0.063	-	5	1 (3 cm)	5	Patients in treatment for substance-use disorders	LC–MS/MS	Cappelle (2018) [73]
	0.063	-	0.063	-	1	Unspecified	≥1	Individuals with self-reported drug use	LC–MS/MS	Cappelle (2017) [74]
	0.11–0.15	-	0.13	0.12	1	4 (1 cm)	4	Child sedated	LC–MS/MS	Kintz (2017) [75]
	0.060–0.099	-	0.075	0.074	3	1–4 (2–6 cm)	6	PM cases, known intake	LC–MS/MS	Krumbiegel (2016) [27]
	1	-	1	-	1	1 (3 cm)	1	Maternal hair sample	LC–MS/MS	Joya (2015) [76]
	0.035	-	0.035	-	1	1 (10 cm)	1	Psychiatric patient	LC–MS/MS	Koster (2014) [77]
	0.013–0.39	-	0.15	0.043	1	3 (1.5 cm)	3	Maternal hair samples, seg. 2–4	LC–MS/MS	Tournel (2014) [78]
	0.037–0.48	-	0.23	0.19	7	1 (5–7 cm)	7	Cases undergoing toxicological investigation, presumed drug use	LC–HRMS	Favretto (2014) [79]
	0.26–0.53	-	0.40	0.40	2	unspecified	≥2	Presumed drug use	LC–HRMS	Favretto (2011) [51]
	0.1–0.3	-	0.2	0.2	9	1 (3 cm)	9	Methadone maintenance therapy	GC–MS	Fucci (2007) [80]
	0.099–0.23	-	0.16	0.16	7	Whole strand	(7)	Methadone maintenance therapy	GC–MS	Lucas (2000) [81]
	0.19–0.67	-	0.39	-	17	1 (3 cm)	17	PM cases, died from overdose	GC–MS	Sporkert (2000) [82]
	0.14–0.67 0.14–0.19 0.031–0.71	- - -	0.18 0.14 0.062	- - -	3	10–29 (2 cm)	29 10 23	Methadone maintenance therapy	GC–MS	Moeller (1993) [83]
	***0.013–0.71***			***0.043–0.40***	***59***		***118 (8)***			***Combined***
**Oxycodone** (Oxymorphone)	≤0.034	-	-	-	2	1 (3 cm)	2	Headache patients	LC–MS/MS	Licata (2016) [20]
	>1.0 (*n* = 2)	-	0.18	-	47	1 (app 4 cm)	47	Individuals ongoing monitoring program	LC–MS/MS	Reisfield (2015) [84]
	**≤*0.034–>1***				***49***		***49***			***Combined***
**Tramadol** (*N*-desmethyltramadol)	0.11–0.83 0.10–0.69 1.4–1.7	- - -	0.27 0.34 1.5	0.26 0.30 1.5	8 8 1	≤4 (0.5 cm)	30 30 4	Controlled single dose study, extensive metabolizers, intermediate metabolizers and primary metabolizers	LC–MS/MS	Johansen (2020) [85]
	- - -	- - -	0.05 0.12 0.24	- - -	14 8 1	Whole strand	(14) (8) (1)	Controlled study, normal CYP2D6 activity, reduced CYP2D6 activity and no CYP2D6 activity	LC–MS/MS	Yu (2018) [86]
	0.093–0.44	0.12–0.44	0.31	0.40	4	2–8 (2 cm)	14	Individuals with kown intake	LC–MS/MS	Verri (2015) [87]
	0.01–0.97	0.037–0.87	-	0.16	75	1 (3 cm)	75	Patients with intake	LC–MS/MS	Madry (2012) [88]
	***0.01–1.7***			**0.16–1.5**	***119***		***153 (23)***			***Combined***
**Tramadol** (O-desmethyltramadol)	0.037–0.70 0.080–0.48 0.064–0.086	- - -	0.30 0.21 0.072	0.29 0.16 0.068	8 8 1	≤4 (0.5 cm)	30 30 4	Controlled single dose study, extensive metabolizers, intermediate metabolizers and primary metabolizers	LC–MS/MS	Johansen (2020) [85]
	0.16–0.22	-	-	-	205	1 (1–6 cm)	205	Individuals undergoing toxicological investigation	LC–MS/MS	Musshoff (2020) [89]
	0.25	-	0.25	-	1	1 (0–2 cm)	1	Patient undergoing treatment	LC–MS/MS	Wang (2019) [90]
	0.11–0.18	-	0.14	0.15	2	1–2 (2–2.5 cm)	3	Suspected DFC and DFSA cases	LC–MS/MS	Wang (2018) [28]
	-	-	0.13 0.04 0.02	-	14 8 1	Whole strand	(14) (8) (1)	Controlled study Normal CYP2D6 activity Reduced CYP2D6 activity No CYP2D6 activity	LC–MS/MS	Yu (2018) [86]
	≤0.050	-	-	-	14	1 (3 cm)	14	Headache patients	LC–MS/MS	Licata (2016) [20]
	0.051–0.11	0.060–0.10	0.077	0.069	4	2–8 (2 cm)	14	Individuals with kown intake	LC–MS/MS	Verri (2015) [87]
	0.021–0.45	-	0.21	0.17	6	Unspecified	≥6	Patients undergoing tramadol therapy	GC–MS	Pinho (2013) [91]
	0.003–0.43	0.021–0.29	-	0.11	75	1 (3 cm)	75	Patients with intake	LC–MS/MS	Madry (2012) [88]
	***0.003–0.70***			***0.068–0.29***	***347***		***≥382 (23)***			***Combined***
**OTHER PHARMACEUTICALS**										
**Atomoxetine** (4-hydroxyatomoxetine)	0.26–2.3	0.33–1.6	0.87	0.66	6	1–3 (1.5 cm)	10	Adolescents + children under treatment	LC–MS/MS	Papaseit (2012) [92]
**Carisoprodol** (Meprobamate)	1.4	-	1.4	-	1	Unspecified	≥1	Suspected carisoprodol abuser	GC–MS	Kim (2005) [93]
**Ketamine** (Norketamine)	≤0.41	-	0.12	0.06	19	1 (3 cm)	19	Individuals with suspected drug abuse	LC–MS/MS	Zhuo (2020) [94]
	0.059–1	-	0.29	0.17	9	Unspecified (5–7 cm)	9	Individuals previously tested positive for ketamine	LC–HRMS	Miolo (2018) [95]
	0.010–1.3	-	0.30	-	526	1 (3 cm)	526	Individuals with ketamine abuse	LC–MS/MS	Leung (2016) [96]
	0.06–0.29 0.09–0.26 0.32–0.80	- - -	0.14 0.19 0.56	- - -	6 8 2	1 (0–6 cm)	6 8 2	Driving relicensing, driving relicensing, medical cases	LC–MS/MS	Salomone (2015) [97]
	0.033–0.20	-	0.12	0.12	3	Whole strand	(3)	Individuals with known drug abuse	LC–MS/MS	Chang (2014) [98]
	0.039–0.13	-	0.069	0.044	2	1–2 (1.5–3)	3	Suspected drug abuser, polydrug intoxication	LC–HRMS	Favretto (2013) [99]
	0.67	-	0.67	-	1	unspecified	≥1	Presumed drug user	LC–HRMS	Favretto (2011) [51]
	0.08–1.1	0.084–0.54	0.28	0.18	10	1 (3 cm)	10	Individuals with drug abuse	LC–MS/MS	Zhu (2011) [100]
	0.045–0.5	-	0.26	0.24	4	Whole strand	(4)	Hair from drug misuse prevention center	LC–MS/MS	Harun (2010) [101]
	0.05–0.84	-	0.33	-	51	≥2 (1–3 cm)	91	Individuals with suspected ketamine abuse	GC–MS	Leong (2010) [102]
	0.005–1.5	-	0.31	0.052	6	Whole strand	(6)	Multi drug abusers	LC–MS/MS	Tabernero (2009) [103]
	0.10–0.38	-	0.21	0.15	3	Full length	(3)	Individuals with a suspected drug abuse	GC–MS	Wu (2008) [104]
	0.043–0.27	-	0.16	0.17	4	Full length	(4)	Individuals with a drug abuse	GC–MS	Wu (2008) [105]
	0.03–0.88	0.043–0.77	0.32	0.30	14	1 (3 cm)	14	Individuals with a ketamine abuse	GC–MS	Xiang (2006) [106]
	***0.005–1.5***			***0.044–0.30***	***668***		**≥686 (20)**			***Combined***
**Ketamine** (Dehydronorketamine)	0.012–0.094	-	0.050	0.045	3	Whole strand	(3)	Individuals with known drug abuse	LC–MS/MS	Chang (2014) [98]
**Methylphenidate** (Ritalinic acid)	0.15–0.53	0.18–0.52	0.35	0.40	7	1–4 (3–14 cm)	17	Illegal use of methylphenidate	LC–MS/MS	Jang (2019) [107]
**Oxcarbazepine** (10-hydroxycarbamazepine)	0.39–0.64	-	0.51	0.50	1	3 (2 cm)	3	PM case, drug addiction	LC–MS/MS	Wang 2017 [40]
	4.7–8.1	-	6.0	5.2	1	3 (2 cm)	3	PM case, psychiatric patient in the past	LC–MS	Klys (2005) [108]
	***0.39–8.1***			***0.50–5.2***	***2***		***6***			***Combined***
**Oxcarbazepine** (Trans-diol-carbazepine)	0.12–0.23	-	0.16	0.13	1	3 (2 cm)	3	PM case, psychiatric patient in the past	LC–MS	Klys (2005) [108]
**Thiopental** (Pentobarbital)	1.0–1.3	-	1.2	1.3	1	3 (1.5)	3	DFSA, proximal segments analyzed	GC–MS/MS	Frison (2003) [109]

**Abbreviations:** -: no information present; AD: antidepressant; combined: summarized MD ratios of pharmaceuticals reported by more than one study presented as ratio range from the lowest and highest reported ratios (based on reported ranges and percentiles) and reported median ratios presented as a range from lowest to highest reported median, if more than one; DFC: Drug facilitated crime; DFSA: Drug facilitated sexual assault; GC–MS: Gas chromatography–mass spectrometry; GC–MS/MS: Gas chromatography–tandem mass spectrometry; LC–HRMS: liquid chromatography–high resolution mass spectrometry; LC–MS: liquid chromatography–mass spectrometry; LC–ECD: liquid chromatography–electrochemical detection; LC–MS/MS: liquid chromatography–tandem mass spectrometry; MD: metabolite-to-drug; no.: number; PCTL: percentile; PM: postmortem; seg.: segments. (A) If only percentiles were reported in a study, this was used as range (B) 5–95 percentile, (C) 25–75 percentile.
